# Men’s and women’s egocentric and allocentric knowledge: The involvement of mental rotation ability and spatial beliefs

**DOI:** 10.3389/fpsyg.2023.1130549

**Published:** 2023-02-23

**Authors:** Laura Miola, Veronica Muffato, Francesca Pazzaglia, Chiara Meneghetti

**Affiliations:** Department of General Psychology, University of Padova, Padova, Italy

**Keywords:** mental rotation, environmental learning, allocentric, egocentric, gender differences

## Abstract

Individual factors can play a relevant role in explaining gender differences in environmental learning in terms of visuospatial abilities and beliefs about spatial abilities, such as stereotypes and growth mindset about navigation ability. In this study, we aim to investigate how mental rotation ability and spatial beliefs interact in the acquisition of egocentric and allocentric spatial knowledge. A sample of 244 participants (140 women) completed individual difference measures, including a mental rotation test (MRT) and questionnaires on gender stereotypes and growth mindsets about navigation ability. Participants then learned a specific route in a virtual environment and performed an egocentric pointing task and an allocentric pointing task. Men performed better in mental rotation and egocentric pointing tasks. Moreover, mental rotation ability predicted both egocentric and allocentric pointing performance; growth mindset predicted allocentric pointing. In general, these results suggest that, despite gender differences in some spatial measures, cognitive abilities and beliefs contribute to supporting environmental knowledge in both men and women.

## 1. Introduction

When navigating, people learn spatial information such as landmarks, routes, and directions, and they create internal spatial representations of the environment (Montello, 1998; Wolbers and Hegarty, 2010). Spatial learning and memory are supported by two types of representation: egocentric and allocentric knowledge. Egocentric knowledge concerns an observer-based or first-person view and defines locations with respect to the body (for example, front-back-left–right). Allocentric knowledge instead adopts an environment-based perspective, disregarding the observer’s current position and defines spatial relations with respect to external objects or cardinal directions – landmark to landmark relations– (Klatzky et al., 1990; Burgess, 2006; van der Ham et al., 2020). Environment knowledge can be represented egocentrically or allocentrically. The latter as result of navigation (i.e., from the person’s point of view is formed a landmark to landmark relation) can allow flexibility to approach several tasks, since it provides fast and route-independent retrieval of landmark locations (Thorndyke and Goldin, 1983; Zhong and Kozhevnikov, 2016). One way to measure the mental representation of an environment from both egocentric and allocentric process, is the pointing task, which can require to judge the direction of landmarks by assessing egocentric knowledge (using the body as a reference to point-to-point direction) and allocentric ones (using landmarks as references to point to the direction of other landmarks).

People vary greatly in their ability to acquire spatial information and mentally represent an environment (Wolbers and Hegarty, 2010). This suggests that individual differences in spatial learning—and among them, gender—can have a relevant role. For example, a meta-analysis considering different tasks such as pointing, retracing routes, orienting with cardinal directions, positioning landmarks, or navigating with verbal instructions showed that overall men outperformed women, with a small to medium effect size (Nazareth et al., 2019). However, if we analyze the allocentric and egocentric knowledge of men and women specifically, the gender differences are less clear. For instance, men seem to perform better than women in tasks that require allocentric or survey knowledge as reported in many studies (e.g., positioning landmarks on a map; Castelli et al., 2008; Burles and Iaria, 2020; van der Ham et al., 2020). However, for egocentric knowledge the differences are less marked where some studies showed that men perform better (in egocentric and allocentric card placing task; e.g., Fernandez-Baizan et al., 2019) but no differences were found in others (e.g., using pointing tasks; van der Ham et al., 2020). Given some mixed results, it is very difficult to take a single view of gender differences in the ability to acquire spatial information and navigation, therefore, further studies are necessary.

Concerning individual differences, there are other factors in addition to gender that seem to be related to environmental learning. Navigation ability is known to be sustained by visuospatial abilities, including the ability to mentally rotate objects (Hegarty et al., 2006; Meneghetti et al., 2014; Muffato et al., 2020). Furthermore, meta-analytic results show that this type of test generates gender differences in visuospatial abilities (Linn and Petersen, 1985). Gender differences in favor of men are well documented, especially in the mental rotation test (Voyer et al., 1995; Pazzaglia and Moè, 2013; Guizzo et al., 2019).

Among individual factors related to navigation ability that can explain some gender differences, there are beliefs about navigation (Nori and Piccardi, 2015; Miola et al., 2021) such as gender stereotype (van der Ham and Koutzmpi, 2022). The literature on gender stereotype has consistently studied stereotype threat (ST; Steele and Aronson, 1995), in which, when a relevant negative stereotype about a minority group is activated, performance on tasks related to the stereotype is reduced. Concerning visuospatial abilities, although some studies find an effect of ST on mental rotation (e.g., Moè and Pazzaglia, 2006; Moè, 2012), a meta-analysis investigating the effects of gender ST showed no significant effect of stereotype manipulation in spatial tasks overall (Doyle and Voyer, 2016). Far fewer studies have considered the effect of gender stereotypes on navigation or environment learning by giving participants instructions activating a stereotype before performing a task. One study found that activating the navigation stereotype enhanced men’s performance, in comparison to the control condition on the navigation task in a virtual environment (stereotype lift; Rosenthal et al., 2012). In a different study, ST affected and men’s and women’s performance especially on a difficult task (Allison et al., 2017). Moreover, one study that measured gender stereotypes by asking people how they thought gender affected spatial performance found that an advantage for men is indicated for both visuospatial abilities (mental rotation) and environmental learning (van der Ham and Koutzmpi, 2022).

Another type of belief recently studied in the spatial cognition domain is the growth mindset, such as the idea that one may enhance his/her intelligence or abilities (Dweck, 2006). To the best of our knowledge, only one study has evaluated the growth mindset in relation to navigational skills showing that people have more fixed beliefs about their ability to navigate than they do about their general intelligence. Furthermore, the growth mindset predicted participants’ navigation ability (He and Hegarty, 2020). Therefore, these results highlighted for the first time the importance of the growth mindset in the spatial domain. However, it is not yet known whether these beliefs can explain gender differences and how they are related to visuospatial abilities and skills in learning an environment (large-scale).

Research on spatial cognition has broadly considered individual factors that can sustain or hamper environmental learning (e.g., gender, visuospatial abilities, and beliefs about spatial abilities). However, there is a lack of knowledge on how these aspects interact in influencing environmental learning and its recall in both men and women.

In this study, we aim to investigate whether individual factors such as visuospatial abilities (in terms of mental rotation) and beliefs about spatial abilities (in terms of gender stereotype and growth mindset) predict independently or in interaction learning an environment in men and women. Specifically, we investigated whether the well-known relationship between mental rotation and environment learning could also be related to the beliefs that people hold, such as gender stereotypes or a growth mindset about their spatial abilities. We specifically focus on pointing tasks as this is one of the ways often used to assess the properties of environmental representations (Mou et al., 2004). We expect interactions between mental rotation and beliefs in spatial abilities with the possibility that the relation between pointing performance and mental rotation ability is positive in those with a higher growth mindset and lower stereotype.

## 2. Method

### 2.1. Participants

This study involved 244 undergraduates (140 females; mean age = 21.84, SD = 2.36; age range 18–29) recruited in exchange for course credits or by word-of-mouth to participate. The study received approval from the University of Padova’s Ethical Committee for Psychological Research (protocol number: 4377). All participants gave their informed consent in accordance with the Helsinki Declaration after being informed of the study’s goals (World Medical Association, 2013). Based on power analyses run with the “pwr” library in R for linear models with six coefficients (gender, mental rotation, growth mindset, gender stereotype and its interactions MRT*growth mindset and MRT*gender stereotype), 114 participants were needed to obtain a power of 0.80 and a small effect size of 0.20, *p* < 0.001.

### 2.2. Materials

#### 2.2.1. Session 1: Visuospatial abilities and questionnaires

Short Mental Rotations Test (sMRT, De Beni et al., 2014). This test consisted of finding two to four objects (3D assembled cubes) that matched the target object in a rotated position (10 items; time limit 5 min). The score corresponded to the number of correct answers. Maximum score is 10.

Gender Stereotype in the Questionnaire on Navigation Ability (GSQ; adapted from Moè and Pazzaglia, 2006). This questionnaire consisted of five items that each described a different spatial task, such as “Finding your way on a map” or “Orienting yourself in a new place.” Using a Likert scale (−3 = women certainly perform better to +3 = men definitely perform better), participants expressed how differently they thought men and women performed on the stated spatial tasks. The aggregate of the answers yielded the final score, running from −15 (favorable stereotypes about women) to +15 (stereotype in favor of men). Cronbach alpha for the current sample: 0.60.

Growth Mindset in Navigation Ability Questionnaire (GMQ; translated from He and Hegarty, 2020). The questionnaire assesses the belief that one’s spatial navigational abilities improve with practice and training *via* a questionnaire. On a 5-point Likert scale, respondents indicated their level of agreement with each of the eight items (1 = not at all to 5 = a lot). The incremental idea was supported in four of the items (for example, “I can always greatly change my ability to navigate.”). The results were flipped for the last four items, which assessed an entity-based perspective on navigational skills or a fixed mindset (example: “I have a specific amount of navigational ability and I cannot modify it.”). The aggregate of the answers yielded the final score. Participants received an explanation of the phrase “navigation ability” before completing the questionnaire (i.e., navigation ability in the environment consists of the ability to move in the environment, reach places, and follow paths). The maximum score is 40. Cronbach alpha for the current sample: 0.89.

#### 2.2.2. Session 2: Navigation learning and recall phase

##### 2.2.2.1. Path-learning phase

A video of a path about 1 km long in a virtual city (modeled with Rhino, Unreal Engine Version 4.21) from a first-perspective (eye height of 160 cm, camera set with a horizontal field of view of 90°) was used in the learning phase (see Miola et al., 2021, for details). The participants watched the video twice, each presentation lasting about 4 min (4 m/s walking speed). During the video presentation, 19 landmarks depicting common buildings of a city were presented.

##### 2.2.2.2. Recall phase

**Figure 1:**
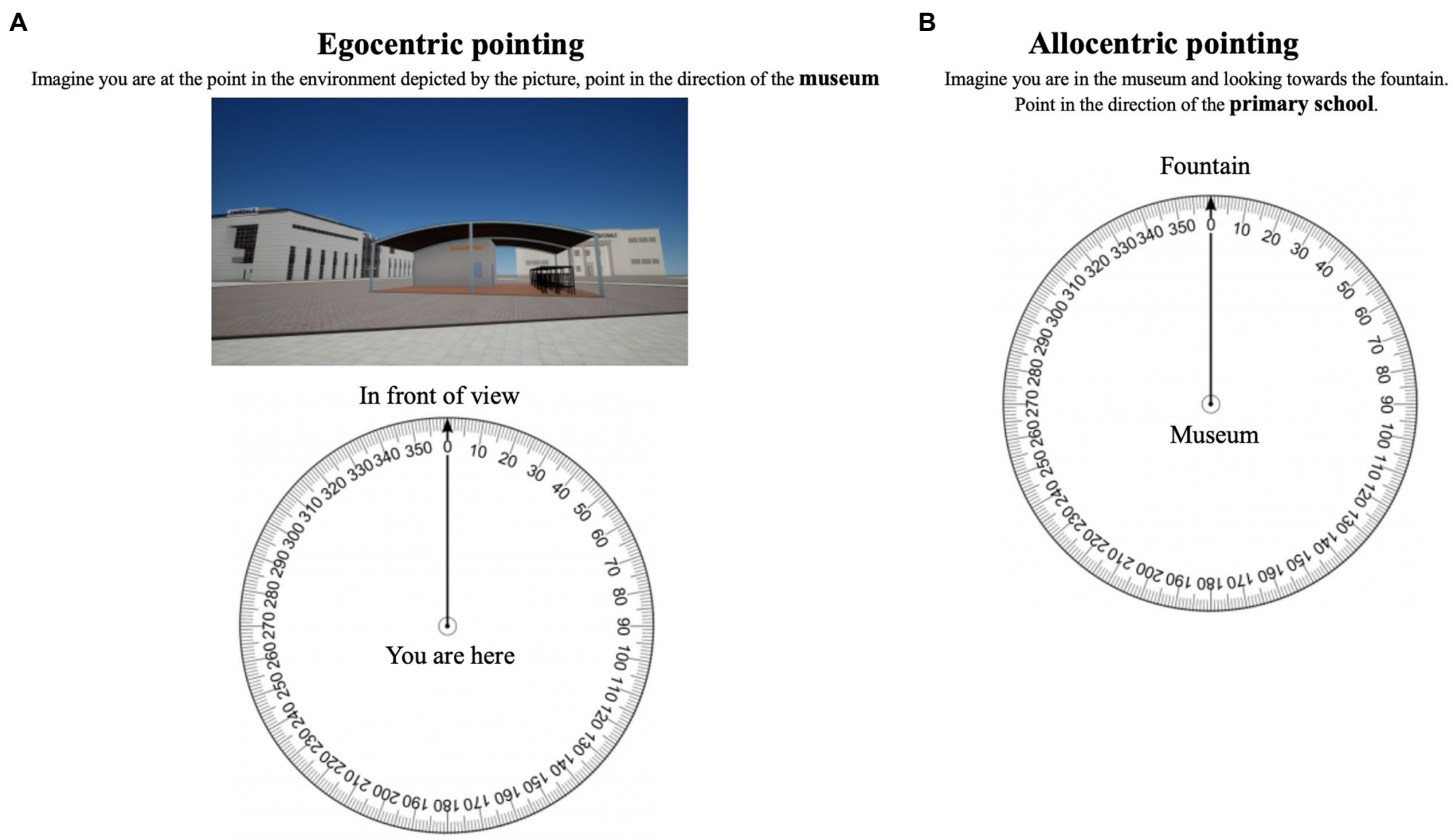
Examples of item for egocentric pointing task **(A)** and allocentric pointing task **(B)**.

Egocentric pointing task. This task consisted of pointing in the direction of a landmark while standing in front of the landmark shown in a screenshot that reflects how the landmark is viewed by the person during the learning phase. Each item had a question at the top of the page, a screenshot of the landmark right below it, and a graduated circle with the answer. There was a total of 6 items, plus one for familiarization. Each participant’s response was compared to the proper direction at the mean of the minimum angles (0–180°; see Figure 1A).

Allocentric pointing task. The task consisted of imagining standing at a given landmark, facing another, and pointing to a third (see Figure 1B) -views not experienced during navigation learning-. For each item, the question was written at the top of a page, and the answer was given using a graduated circle. There was a total of 6 items in all, plus one for familiarization. Each participant’s response was compared to the proper direction at the mean of the minimum angles (0–180°). The main difference between the egocentric and allocentric tasks consists of the fact that in the first one is displayed the image of the landmark in which the person is looking at, while in the allocentric task no image is shown but requires imagining all the landmarks.

### 2.3. Procedure

Individually, participants attended two online sessions (45-min each) and signed an informed consent form. The experimenters introduced themselves to the participants in Zoom and gave them a link to Qualtrics during the first session. In a random order, participants completed questionnaires including the growth mindset and completed the visuospatial tests on mental rotation ability. The experimenter reconnected with the participants during the second session *via* the Zoom platform and gave them another Qualtrics link. Participants watched a video of a virtual environment twice to memorize the route before performing the following tasks in random order: egocentric pointing (with random presentation of things), allocentric pointing (with random presentation of items). At the end of the experiment, participants responded to the gender stereotype questionnaire. Participants in sessions 1 and 2 completed other tasks and questionnaires not considered here because they were not related to the scope of this study.

### 2.4. Statistical analysis

First, we examined gender-related differences (see Table 1).

**Table 1:** Descriptive statistics of total sample, and divided for men and women.

	Total sample *N* = 244		Women *N* = 144		Men *N* = 104			
	*M*	SD	*M*	SD	*M*	SD	*t*	*p*
Growth mindset (GMQ)	27.52	4.75	27.39	4.57	27.69	4.99	−0.48	0.63
Gender stereotype (GSQ)	0.35	0.45	0.36	0.43	0.34	0.47	0.37	0.70
**Mental rotations Task**	**5.25**	**2.57**	**4.70**	**2.45**	**6.00**	**2.54**	**−4.01**	**<0.001**
Pointing egocentric*	**61.61**	**22.80**	**64.17**	**22.65**	**58.16**	**22.65**	**2.04**	**0.04**
Pointing allocentric*	78.30	28.80	79.04	28.71	77.30	29.02	0.46	0.64

In bold the significant gender differences. *Degree of error.

Then we carried out two generalized linear regression models on egocentric and allocentric pointing tasks. In both models, independent variables (predictors) were added in the following order: gender (Step 1), mental rotation test (MRT; Step 2); beliefs about spatial abilities (gender stereotype and growth mindset; Step 3); interactions between mental rotation ability and beliefs about spatial abilities (MRT*growth mindset + MRT*gender stereotype; Step 4). An AIC-based stepwise approach was used to find the best model with a lower AIC (Akaike Information Criterion; Akaike, 1973). The same procedure was carried out for each navigation task (e.g., pointing egocentric and allocentric). Tables 2, 3 showed the models with the better and lower AIC for each score in pointing task (egocentric and allocentric).

**Table 2:** Results from the best-fitting model for the Egocentric pointing after model selection process.

Predictors	Egocentric pointing			
	Estimates	Std. Beta	CI	Value of *p*
(Intercept)	0.07	0.07	−0.09 to 0.24	0.382
Gender	−0.17	−0.17	−0.43 to 0.09	0.189
MRT	−0.18	−0.18	−0.31 to −0.05	**0.006**
*R*^2^	0.047			
AIC	687.65			

Statistical significance in bold.

**Table 3:** Results from the best-fitting model for the Allocentric pointing after model selection process.

Predictors	Allocentric pointing			
	Estimates	Std. Beta	CI	Value of *p*
(Intercept)	−0.02	−0.02	−0.18 to 0.15	0.839
Gender	0.04	0.04	−0.22 to 0.30	0.760
MRT	−0.19	−0.19	−0.31 to −0.06	**0.004**
Stereotype	−0.09	−0.09	−0.22 to 0.03	0.134
Growth mindset	−0.16	−0.16	−0.28 to −0.04	**0.011**
*R*^2^	0.066			
AIC	686.83			

Statistical significance in bold.

## 3. Results

Descriptive statistics and gender differences are shown in Table 1. A statistically significant gender difference emerged for mental rotation ability (*t* = −4.01, *p* < 0.001, Cohen’s *d* = −0.52, 95% CI [−0.78–0.26]), and for egocentric pointing (*t* = 2.04, *p = 0*.04, Cohen’s *d* = 0.26, 95% CI [0.01–0.52]) where men outperformed women.

We ran two models following the model selection procedure explained in the statistical analysis section relative to the dependent variables: (a) the egocentric pointing task, and (b) the allocentric pointing task (see Tables 2, 3). For egocentric performance, the AIC indices were: m1(+gender) = 693.25; m2(+MRT) = 687.65; m3(+growth mindset, +gender stereotype) = 689.14; m4(+MRT*growth mindset, +MRT*gender stereotype) = 692.84, therefore m2 resulted to be the best model.

For allocentric performance, the AIC indices were: m1(+gender) = 693.24; m2(+MRT) = 687.65; m3(+growth mindset, +gender stereotype) = 689.15; m4(+MRT*growth mindset, +MRT*gender stereotype) = 692.84, therefore m3 resulted to be the best model.

For the egocentric pointing task, the best-fitting model (m2) included gender and mental rotation as predictors. Results showed a statistically significant effect of mental rotation (*B* = −0.18, CI [−0.31, −0.05, *p* = 0.006]). The total variance explained was *R*^2^ = 0.05.

For the allocentric pointing task, the best-fitting model (m3) included gender, mental rotation and beliefs about spatial abilities (growth mindset and gender stereotype). The results showed a statistically significant effect of mental rotation (*B* = −0.19, 95% CI [−0.31, −0.06, *p* = 0.004]) and growth mindset (*B* = −0.16, 95% CI [−0.28, −0.04, *p* = 0.011]). The total variance explained was *R*^2^ = 0.07.

## 4. Discussion

In this study, we aimed to investigate differences in spatial performance (mental rotation, egocentric and allocentric task) and beliefs about spatial abilities (growth mindset and gender stereotype) between men and women. Moreover, we investigate the role of mental rotation ability and beliefs about spatial abilities (growth mindset and gender stereotype) in the relationship with egocentric and allocentric pointing performance respectively, in men and women. Specifically, we investigated whether the well-known relationship between mental rotation ability and performance in environmental learning is characterized by an interplay with beliefs about spatial abilities.

First of all, the results confirm differences in favor of men on mental rotation task (e.g., Guizzo et al., 2019) corroborating that mental rotation test has seen more extensive advantages of men (for a review see Voyer et al., 1995). Furthermore, we found an advantage for men in the egocentric pointing in line with previous studies (e.g., Fernandez-Baizan et al., 2019). These results highlighted the heterogeneity of gender differences, showing that gender differences are found not only or more frequently in allocentric tasks but also in egocentric tasks. In light of these considerations, spatial cognition research should focus on other factors related to spatial acquisition, such as beliefs about spatial abilities, because factors other than the type of knowledge could explain spatial navigation performance in men and women. In the present study, we investigated the beliefs of growth mindset and gender stereotypes with respect to spatial abilities (which recently emerged as important factors in explaining spatial acquisition) but no difference emerged in our results.

Subsequently, after accounting for gender, we investigated the role of mental rotation ability and beliefs about spatial abilities (both separately and in interaction) in the acquisition of egocentric and allocentric knowledge in men and women. The results showed an involvement of mental rotation for egocentric knowledge. After accounting for gender, people with higher score in the mental rotation task performed better (made fewer errors) in egocentric pointing, confirming the role of rotation ability in the creation of a mental representation of the environment (e.g., Meneghetti et al., 2014). Furthermore, for allocentric pointing performance, we found an involvement of mental rotation and growth mindset; that is, after accounting for gender, people with higher score in the mental rotation but also higher levels of growth mindset performed better (made less errors) in allocentric pointing. In summary, while the results showed the involvement of mental rotation in both egocentric and allocentric tasks, the growth mindset emerged only in the allocentric task. These findings suggest that the set of beliefs about the malleability of our spatial abilities and competence may play an important role in the creation of allocentric knowledge. Therefore, the contribution of personal beliefs emerged in addition to mental rotation skills in the allocentric knowledge (landmark to landmark based) after navigation (person point of view based) in which there is a change of perspective in knowledge formation (and flexibility in knowledge formation).

Finally, after controlling for gender, we found no interaction between mental rotation ability and beliefs about spatial abilities suggesting that they both sustain in parallel mental representation. However, their interactions should be further investigated in future studies.

The lack of an effect of gender stereotype is in line with work that has shown no effect of gender stereotype in spatial performance when manipulated (Doyle and Voyer, 2016). Although gender stereotype seems important for spatial abilities (e.g., mental rotation; Moè and Pazzaglia, 2006), its role seems less relevant for large-scale ones.

To conclude, beliefs about one’s abilities, especially growth mindset in navigation ability, are aspects that should be considered for the implementation of spatial skills enhancement training.

Concerning the limitation of the study, it did not consider other individual factors such as emotional aspects that can play a role in explaining spatial knowledge or gender differences. Specifically, it would be interesting to study the relationship between emotional aspects like spatial anxiety (or affective states) and beliefs in one’s ability, in predicting the acquisition of spatial knowledge (Lourenco and Liu, 2023). Future studies could investigate whether different levels of anxiety or emotions can moderate the relationship between beliefs and spatial performance.

In conclusion, this study is the first to investigate mental rotation ability and beliefs about spatial ability (growth mindset and gender stereotype) with egocentric and allocentric spatial knowledge, highlighting that role of mental rotation for both types of spatial knowledge and the role of the growth mindset in sustaining especially allocentric knowledge. Therefore improving and increasing awareness of beliefs about growth mindset could be an important point to support people’s ability to navigate in everyday life.

## Data availability statement

The raw data supporting the conclusions of this article will be made available by the authors, without undue reservation.

## Ethics statement

The studies involving human participants were reviewed and approved by the Ethical Committee of University of Padova (protocol number: 4377). The patients/participants provided their written informed consent to participate in this study.

## Author contributions

LM, VM, FP, and CM contributed to the design and implementation of the research, to the analysis of the results and to the writing of the manuscript. All authors contributed to the article and approved the submitted version.

## Funding

This research did not receive any specific grants from funding agencies in the public, commercial, or not-for-profit sectors.

## Conflict of interest

The authors declare that the research was conducted in the absence of any commercial or financial relationships that could be construed as a potential conflict of interest.

## Publisher’s note

All claims expressed in this article are solely those of the authors and do not necessarily represent those of their affiliated organizations, or those of the publisher, the editors and the reviewers. Any product that may be evaluated in this article, or claim that may be made by its manufacturer, is not guaranteed or endorsed by the publisher.
